# Advanced Design of Fiber-Based Particulate Filters: Materials, Morphology, and Construction of Fibrous Assembly

**DOI:** 10.3390/polym12081714

**Published:** 2020-07-30

**Authors:** Seojin Jung, Jooyoun Kim

**Affiliations:** 1 Department of Textiles, Merchandising and Fashion Design, Seoul National University, Seoul 08826, Korea; wjdwls04079@snu.ac.kr; 2 Research Institute of Human Ecology, Seoul National University, Seoul 08826, Korea

**Keywords:** air filter, performance, resistance, multifunctional, morphology, test parameter

## Abstract

With increasing air pollution and sporadic outbreaks of epidemics, there is ramping attention on the filtration devices. The main constituents of airborne pollutants are particulate matters of solid particles, liquid aerosol, bioaerosol/bio-droplets, and gas/vapor. With the growing demand for high-performance filters, novel materials and functionalities are being developed applying advanced technologies. In this paper, recent developments of fiber-based particulate filters are reviewed, with a focus on the important performance parameters and material properties. Trends in technology and research activities are briefly reviewed, and the evaluative measures of filtration performance are reported. Recent studies on the advanced filter materials are reviewed in the aspect of polymers and the fabrication process of fibrous assembly. The characterization method including 3D modeling and simulation is also briefly introduced. Multifunctional filters such as antimicrobial filter and gas and particulate filters are briefly introduced, and efforts for developing environmentally sustainable filters are noted.

## 1. Introduction

With increasing air pollution and sporadic outbreaks of epidemics, there is ramping attention on the filtration devices. Previously, air-purifying respirators (APR) had been worn mainly by the first responders and industrial workers for respiratory protection from the exposure to airborne particles and gaseous matters. Nowadays, the protective facemasks became more an essential for public health in hazy days and virus outbreak.

The main constituents of airborne pollutants are particulate matters of solid particles, liquid aerosol, bioaerosol/bio-droplets, metal fume, gas, and vapor (Table 1). Particulate matters (PM) that are categorized by the size of particles and PM_2.5_, of which the particle size is smaller than 2.5 μm, are reported to be especially harmful because they can reach the thoracic region and blood stream, causing respiratory and cardiovascular diseases [1]. The associated risk can be exacerbated when pathogenic microbials including bacteria and virus are present in the air [2]. Coughing, sneezing, and talking can generate microbial aerosols and droplets in the air, potentially carrying infectious diseases. Thus, the role of air purifying devices becomes more important in the occurrence of viral pandemics such as COVID-19, SARS, H1N1 influenza, and in the secondary bacterial infections associated with viral epidemics.

As protective tools, filtration devices such as APR and heating, ventilation and air conditioning (HVAC) systems are used. For particulates, fiber-based filter media are generally used. A commonly used fibrous filter is made from nonwoven media made by meltblowing, electrospinning, air-laid, and the combination of different webbing processes [8,9,10]. For gaseous matters, activated carbon is commonly applied to purifying cartridges or face masks for a quick capture of gas and vapor molecules [11,12,13]. Various airborne pollutants are summarized in Table 1 with information of their general size ranges.

**Figure 1:**
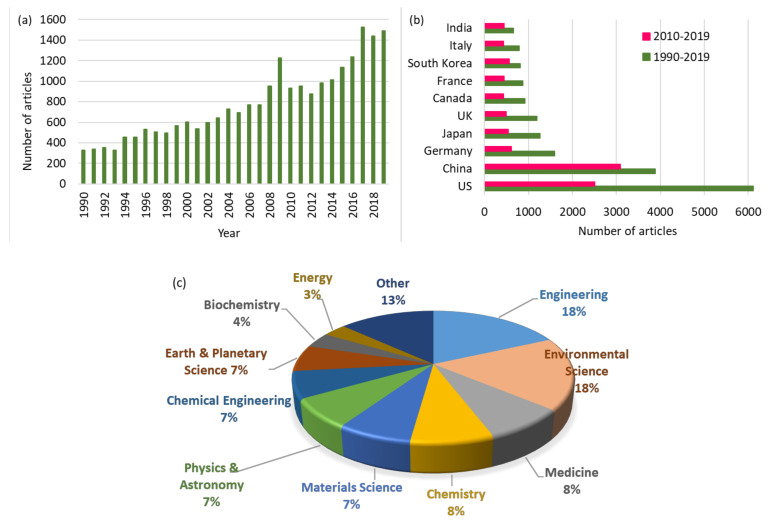
Research trends surveyed with key words of ‘filter’ and ‘air’ in the years 1990–2019. (**a**) Number of journal articles; (**b**) Published articles by country; (**c**) Articles by subject area. The research analytics are sourced from Scopus.com.

With the growing demand for high-performance filters, increasing research efforts are being made on understanding the filtration mechanism and developing novel filter materials. Figure 1 shows the survey results of a number of published journal articles in recent 20 years, with keywords of ‘air’ and ‘filter’ in the Scopus analytics site. Overall, a significant academic endeavor has been invested globally to overcome the serious airborne pollution from various research sectors. Of particular attention is the increased number of published articles from China and South Korea in recent ten years, reflecting the striving effort for responding to serious air pollution in this region.

**Figure 2:**
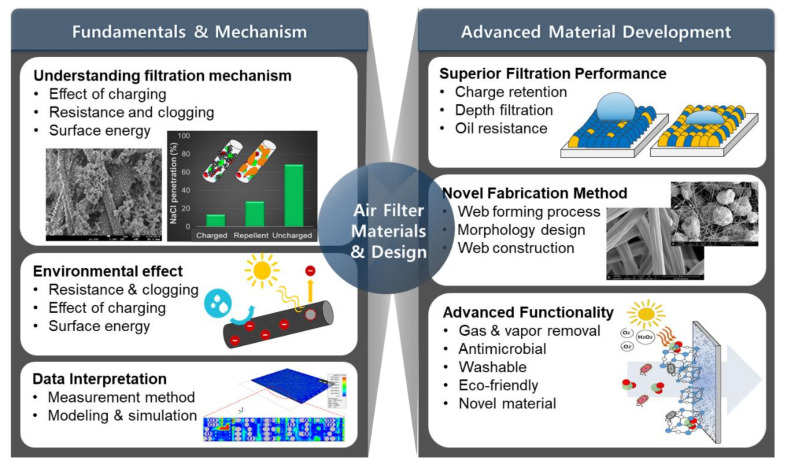
Research topics related to air filters.

In this review, recent developments of fiber-based particulate filters are reviewed, with a focus on the important performance parameters and material properties. Research trends are briefly reviewed, and the evaluative measures of filtration performance are reported. Recent studies on the advanced filter materials are reviewed in the aspect of polymers and the fabrication process of fibrous assembly. The characterization method including 3D modeling and simulation is also briefly introduced. Multifunctional filters that have an antimicrobial effect and gas removal function are briefly introduced. Finally, efforts for developing environmentally sustainable filters are noted. Overall, the research topics reviewed in this paper are shown in Figure 2.

## 2. Filtration Performance of Fiber-Based Filters for Particulates

### 2.1. Fiber-Based Filtration Mechanism

**Figure 3:**
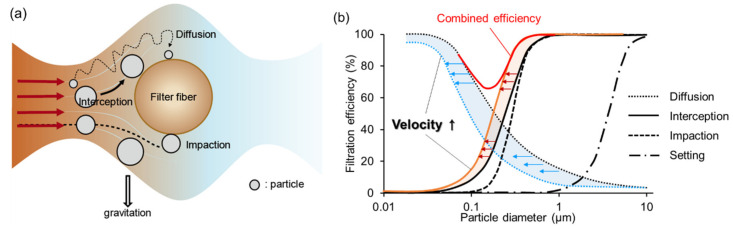
Illustration of the particle capture mechanism. (**a**) Mechanical capture of particles by a single fiber [14]; (**b**) Combined effect of the mechanical capture mechanism on the overall filtration efficiency [15,16,17,21].

Airborne particles and aerosol can be mechanically captured by a single fiber through the mechanisms of interception, inertial impaction, diffusion, and gravitational settling [14], as illustrated in Figure 3a. Interception occurs when a particle follows the air stream line and makes a physical contact onto the media fiber. Inertial impaction occurs when a heavy particle cannot adjust to the abrupt changes in the air stream direction near the fiber, and due to the inertia, the particle continues along the original path and contacts the fiber. The inertial impaction works better at the high face velocity [15,16,17]. Diffusion is effective for small particles (<0.1 μm), and in this case, particles make a random Brownian motion and collide with the fibers. The diffusion mechanism is more effective for small particles at the slow air stream [15]. The gravitational sediment occurs for large and heavy particles that settle by gravity, and this gravity effect is negligible for particles smaller than 0.5 μm [14].

Overall, mechanical filtration efficiency is the result of a combined effect of those capture mechanisms. Very small particles less than 0.1 μm are primarily captured by the diffusion mechanism, and large particles greater than 0.4 μm are captured by interception and inertial impaction [14,18]. Generally, the particles between 0.1–0.4 μm are relatively less efficient to capture, and the most penetrating particle size (MPPS) range varies depending on the face velocity and fiber size [14,15,18] (Figure 3b).

When a fiber is electrostatically charged, electrostatic attraction and induced polarization additionally contribute to the capture of particles [19,20]. Electret fibers are generally composed of charges of both signs, and charged fibers effectively attract both the oppositely charged particles and the uncharged particles. For uncharged particles, fiber charges induce the instant dipoles on the particles when particles come close to the charged fiber surface, attracting the particles [20]. Commercially available filters are mostly electrically charged called electrets, and the use of electret filters significantly improves the particle collection efficiency, especially in the MPPS range.

### 2.2. Measurement of Particulate Filter Performance

#### 2.2.1. Testing Parameters

Filtration performance is generally evaluated by the penetration of solid particles or oily aerosol at the specific flow rate and mass concentration of aerosol. For example, filtering face masks that are certified by the US National Institute for Occupational Safety and Health (NIOSH) 42 CRF Part 84 are tested against either NaCl particles of which count the median diameter (CMD) of 0.075 ± 0.02 μm or dioctyl phthalate (DOP) aerosol with CMD of 0.185 ± 0.02 μm, at 85 LPM of air flow [22,23]. The size of test particles is in the range of MPPS, and the charges of particles are neutralized to zero in the Boltzmann charge distribution, to make the test condition the worst case scenario.

Between the solid and oily aerosol, oily aerosol makes the filter efficiency deteriorate more rapidly in general, because the oily liquid easily spreads on the fiber surface, quickly masking the charged sites [24,25]. The air flow influences the air resistance or pressure drop of the test sample. More specifically, the resistance is proportional to the air face velocity [26], where the face velocity is calculated from the air flow rate divided by the test area. Thus, when evaluating the resistance of a filter, the face velocity of the test condition should be considered, as the face velocity is directly correlated with the resistance [26,27].

#### 2.2.2. Filtration Performance and Service-Life

**Figure 4:**
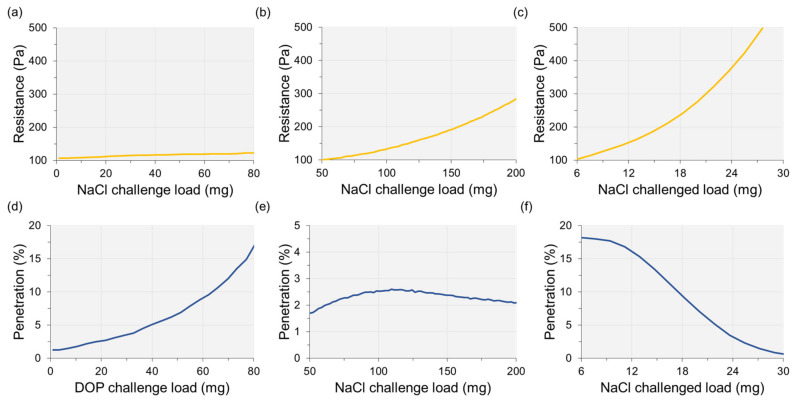
Resistance and penetration of filter materials with mass loading of solid and oily aerosols at 85 LPM. (**a**) Resistance of oily aerosol; (**b**) Resistance of solid aerosol for a loosely packed electret filter; (**c**) Resistance of solid aerosol for a dense filter; (**d**) Penetration with oily aerosol; (**e**) Penetration with solid aerosol for a loosely packed electret filter; (**f**) Penetration with solid aerosol for a dense filter. Tests were conducted by the authors.

Generally, the percentage of particle penetration (100%—% efficiency) and the resistance are used as main indicators of filter performance. Filtration efficiency is measured either by the instantaneous penetration at the initial aerosol exposure or the maximum penetration during the mass loading of the aerosol. Figure 4 illustrates the loading behavior of different aerosol types and filters. For the oily aerosol, penetration generally continues to increase with the increased mass loading. The resistance does not change much, as the oily liquid spreads over the surface instead of clogging the pores [25,28,29]. For the solid aerosol, the loading trends can differ by the filter characteristics. For the porous electret filter that allows depth loading, penetration initially increases as the charged surface is masked by the deposition of solid particles; then after reaching the maximum penetration, the penetration begins to decrease as the solid particles clog the pores of the filter material [30,31,32]. The resistance may build up more quickly after reaching the maximum penetration, as the pores of the filter are clogged. For a filter material that the mechanical filtration mechanism is dominant, it is likely that the filter begins to get clogged in the early stage of aerosol loading; in this case, penetration keeps decreasing from the initial loading, and the resistance builds up steeply from the beginning.

As the loading trend varies by the challenge agent, the service life of a filter material can be also influenced by the aerosol type. It is likely that the solid aerosol limits the service life of a filter material by the build-up of resistance, whereas the oily aerosol limits the service life by the loss of filtration efficiency. To extend the service life against solid particles, the filter material needs to be designed to lower the resistance by allowing the depth loading. For the oily aerosol, the wettability of filter material can be modified to deter the quick spreading of the oily liquid; for this purpose, a hydrophobic/oleophobic treatment can be beneficial for extending the effective service life against the oily aerosol [30,33,34]. The oleophobic filter material can be applied to the NIOSH R- and P-type filters that require a certain level of efficiency during 200 mg of DOP loading.

**Figure 5:**
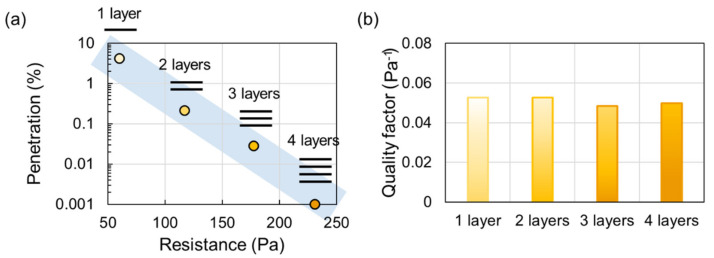
Effect of layers on filtration performance. (**a**) Effect of layers on penetration and resistance; (**b**) Effect of layers on quality factor. Tests were conducted with NaCl particles at 85 LPM by the authors.

Generally, a high filtration efficiency is traded off by a high resistance, and the respirator users are forced to compromise the protection performance with the breathing comfort. Thus, the design for superior filtration should be directed to have a high collection efficiency at a low resistance. To account for both efficiency and resistance, the concept of quality factor (QF) is employed, where QF = −[ln(penetration)]/resistance. From the equation, a higher QF indicates a higher efficiency at the unit resistance. The QF can be used as an inherent quality of filter media; that is, a multi-layer construction made of the same filter media would have a similar QF as the single layer, while the multi-layers may have a higher filtration efficiency and a higher resistance than a single layer. A typical example of the layering effect on performance and quality factor is shown in Figure 5.

## 3. Materials for Superior Filtration

### 3.1. Charge Retention Capacity of Electret Filters

For commercial filters, polypropylene (PP) is commonly used for meltblown (MB) nonwoven materials. PP is a nonpolar material with a low surface energy (27–33 mJ/m^2^). Its melt is used to form fibrous web, and the meltflow index (MI) of PP resin should be carefully chosen for proper processability. The PP MB web is often electrostatically charged to enhance the filtration efficiency. The charges held in PP stay relatively longer, as they are inert to conductive molecules such as water. Such a dielectric material that retains electric charges is called an electret [35,36].

The main charging techniques applied to electret filters include corona charging, tribocharging, and electrospinning [37]. Corona charging is commonly done to PP meltblown, where the polymer is exposed to corona discharges produced by a wire electrode [38]. Corona charging generates both the hetero- and homo-charged electrets. Hetero-charging is the result of orientation of dipoles and migration of free charges, while homo-charging occurs by charge injection from the ionized air [39]. A nonpolar polymer with a low ohmic conductivity is reported to make a homo-electret, giving a stable electret [39].

Tribocharging is a process where materials in dissimilar electronegativity contact each other and produce charges in the opposite signs [40]. Tribocharged webs display the surface charges in the opposite polarities, both positive and negative, behaving as bipolar materials [37]. Generally, corona and tribocharging processes produce slight dipole charges and high space charges, and they show gradual charge dissipation, resulting in the lowered filtration efficiency [41].

Electrospinning is a nanofiber fabrication technology that provides in situ charge injection prior to the fiber formation, generating the space charges of electrospun fibers with a great depth of energy level [41,42]. The advantage of this technique is that it produces the charged fibers in a one-step process, where the polymer solution is extruded through an electrode to a collector in an electric field, and it can produce nano-sized fibers that could enhance the mechanical filtration performance [43]. However, the enhanced mechanical filtration may lead to a higher resistance, thus the process parameters of electrospun filter media need to be carefully adjusted.

In general, electret filters as opposed to mechanical filters exhibit higher quality factor with the benefit of electrostatic filtration mechanism, but this benefit can be limited when the electret charges are deteriorated by the environmental aging. Charge retention ability is reported to depend on the electrical properties of polymers such as conductivity and dielectric constants [37,39,44]. From a study by Lee and Kim [45], PP, poly(acrylonitrile) (PAN), and poly(vinylidene fluoride) (PVDF) which have different dielectric constants and moisture absorption properties were compared for the charge stability and its impact on filtration performance, when treated under high temperature (120 °C) or high humidity (25 °C, 90% RH). The effect of moisture-aging on charge decay was highest in the order of PAN > PVDF > PP, corresponding to the order of moisture absorption capacity. For thermal treatment, charge decay appeared larger in the order of PVDF > PAN > PP, showing that the material with higher dielectric constant was more vulnerable to charge loss, leading to deterioration of filtration performance. The effect of thermal aging on the filtration performance appeared significantly larger than the effect of humidity aging for all filter materials from this study [45].

The charge retention capacity of a dielectric polymer is generally associated with the electrical conductivity and the energy level of localized trap sites [45,46,47,48,49,50]. The dielectric property is affected by the electron hopping at each energy level, where the band gap energy becomes important [49,50,51]. According to Ravichandran et al. [50], the dielectric constant is inversely proportional to the square of the band gap energy [50,51]; thus, the material with a higher dielectric constant would have a smaller band gap energy, and the loss of charge can be much easier for this material. Similarly, the material with a high electrical conductivity would have a smaller band gap [49]. From this perspective, a nonpolar polymer would be a good candidate of electret material due to its low electrical conductivity [52,53]. The material with a high trap energy can hinder the charge transport to the conduction band, demoting the charge decay [45,46].

The mechanism of charge decay is generally explained by the relaxation of polarized state of dielectrics and the spatial distribution of dipoles and free charges [54,55,56,57]. Environmental factors such as heat, moisture, or solvent exposure can cause electric conduction, facilitating the charge carrier mobility, and leading to charge dissipation [57,58,59,60,61]. In Cho et al.’s study [36] where the charge retention of polycarbonate electrospun web was investigated, the charge decay was aggravated at above the glass transition temperature (*T*_g_). It was reported that at above *T*_g_, the segmental depolarization of oriented dipoles occurred by the facilitated molecular motion, and this led to the accelerated charge decay.

### 3.2. Advanced Electret Filter Materials

Efforts have been made to increase the surface potential of electret filter materials and to enhance the long-term service performance. PVDF, as a dipolar material, has been studied to apply as an effective electret filter [41,42,45,62,63]. It was expected that, when PVDF was electrospun, the crystal phase of PVDF molecules would be transformed from the nonpolar α-phase to the polar β-phase due to the spontaneous polarization upon electrospinning, and this β-phase conversion would maximize the dipole moment [41,42]. The surface potential and the charge stability of PVDF electrospun web was further improved by the addition of polytetrafluoroethylene (PTFE) nanoparticles that acted as a charge enhancer [41]. In this study, the charge stability was measured by the thermally stimulated discharge current (TSDC), and it was proposed that the increased amount of PTFE particles was in correlation with the dipole polarity, where it was associated with the increased depth of energy for charge trapping [41]. For the long-term serviceability of electret filters, it is desirable to maintain the stable charges and the mechanical properties of filter material, even in a humid and heated condition [64,65].

In addition to making a long-surviving electret, magnetic force was used by incorporating magnetic particles in fibers [66,67,68]. In a study by Kim et al. [69], poly(vinylpyrrolidone) (PVP) was electrospun, dispersing Fe_3_O_4_ magnetic nanoparticles in the pre-spinning solution. When the produced web was magnetized by an external magnetic field, the collection efficiency of metal oxide particles (Fe_2_O_3_), one of the major components of subway dust, was greatly enhanced [69]. Zhao et al. [54] incorporated various kinds of negative ions including Al_2_O_3_, SiO_2_, MgO, Fe_2_O_3_, Na_2_O, TiO_2_, and FeO into the PVDF electrospun fibers. The produced web was capable of releasing negative ions and showed an effective filtration performance. Likewise, the electrostatic or magnetic attraction in addition to the mechanical filtration eventually improved the quality factor of the filters [70,71,72,73].

In general, the filtration performance of electret filters decreases more rapidly against the oily aerosol than the solid aerosol, because the immediate spreading of oily liquid on the fiber surface masks the surface charges of the filters [25,29,30]. To prevent the rapid masking of charges, the surface of electret filters was modified for oil resistance, and the effect of hydro/oleophobic treatment on filtration efficiency was investigated [24,30,33,55,74]. The surface energy of filter media was lowered by fluorination of the surfaces [24,30,33,55,74], and the resulting web showed superhydrophobicity or superoleophobicity. When the immediate wetting by the oily aerosol was deterred, the collection efficiency against dioctyl phthalate (DOP) was maintained longer with the continued loading of aerosol [30], while the instantaneous efficiency was not significantly different [24]; with a submicron layer of coating, the resistance was not affected. For some cases, oleophobic coating decreased the pore size of the filter media, and it led to the increase of instantaneous filtration efficiency against both oil and solid aerosols; however, the improved efficiency appeared to be the result of enhanced mechanical filtration by the blocked pores, and this would increase the resistance, giving an adverse effect [74].

The collection system of oil aerosol was explained by the collision and drainage mechanism from the study by Wei et al. [33]. In this study, a double layered structure with superoleophobic and oleophilic layers was examined, and the collection efficiency against oily aerosol was found to be more effective when the superoleophobic layer was positioned in the second layer. In this sequence of construction, the oily aerosol collected in the first oleophilic layer was blocked by the superoleophobic second layer [33]. In contrast, when the superoleophobic layer was positioned in the first layer, the oil collected by this layer was easily transported into the oleophilic layer, penetrating to the down flow.

A hybrid approach of fabricating a stable electret web was made by Jiang et al. [55]. In this study, poly(vinyl butyral) (PVB) with a high electrical resistivity was electrospun with the dispersion of silicon nitride (Si_3_N_4_) particles with a high dielectric constant. The Si_3_N_4_ acted as a charge enhancer, and the resulting web showed a high surface potential. When PVB was oleophobized by the addition of fluorinated polyurethane then doped with Si_3_N_4_ particles, the resulting oleophobic electret composite displayed the enhanced charge stability with a high surface potential. Overall, strategies for making a stable electret with a high surface potential have been discussed as: (1) hybridization of materials with different dielectric constants [41,55,69]; (2) reduction of surface energy to resist oil spreading or moisture absorption [24,30,33,74]; (3) reduction of electrical conductivity by using polymers with a high resistivity [45,55]. Table 2 summarizes the strategies for fabricating high-performing electret filters.

## 4. Fibrous Assembly for Advanced Filtration

### 4.1. Effect of Fiber Morphology on Filtration Performance and Resistance

As particles are captured by contacting on a single fiber, not by sieving, the fibrous structure with a high specific area and with long air pathways are beneficial for filtration. Most of the commercial filtering facepieces are made of meltblown web; as its open structure induces the tortuous air pathway, and allows breathability while capturing particles. As a versatile technique to design the submicron to micron fibers with various morphological structures [24,110,111,112,113,114], electrospinning has been employed [14,24,36,41,47,56,69,74,83,85,89,93,95,97,104,106,108,115,116,117]. Electrospun nanofibers can be advantageous for capturing small particles due to their high specific surface area and the inherent initial charges resulting from the electric field-driven process. However, nanofibers with a high packing density may result in a high resistance that leads to an earlier clogging [30,31,97,106]; thus, design parameters including fiber size, solidity, thickness, and basis weight should be prudently chosen to satisfy both filtration efficiency and resistance.

For submicron fibers with extensively interconnected pores, the aerodynamic behavior of airflow around the fibers is correlated with the mean free path of air molecules [96,118]. Theoretically, the mean free path of air molecule is 65.3 nm at 25 °C and 1 atm; when the fiber diameter is near 65.3 nm, the drag force of air stream was significantly lowered due to the slip flow effect [119]. When the slip effect was tested with a fiber diameter range of 60–100 nm with an aperture size (distance between adjacent fibers) of >3.5 μm, the air resistance decreased as the fiber diameter decreased [118]. Thus, efforts were made to further decrease the fiber size, and 2D nanonets with interlinked nanowires were made by the electrospinning process [42,81,82,83,104]. The nanonets were formed by adding the ionic liquids such as LiCl in the spinning solution to increase the conductivity, and the produced nanonets with ~20 nm fibers showed the enhanced slip effect, where the fiber size was much smaller than the mean free path of air (66 nm) [82]. Several polymers of nanofiber nets were fabricated including polyamide 6 [42,104], polyamide 56 [83], polyamide 66 [105], PAN [42], PVDF [42], polyurethane (PU) [42,82], poly(m-phenylene isophthalamide)/PU [86], etc., and those nanonets showed enhanced mechanical filtration performance. When nanonets were electrospun onto a non-planar geometry such as a rippled fibrous scaffold, the quality factor and the dust holding capacity were increased due to the increased surface area [104]. As an effort to enhance the particle filtration, metal organic frameworks (MOFs) were incorporated in nanofibers [78]. The porous structure and large surface area of MOFs not only enhanced the mechanical capturing of PM, but also the charges of metal ions enhanced the electrostatic interactions with particles [80,120].

While a high quality factor has been demonstrated for nanofibrous filters [24,30,121,122,123], those studies tested only the instantaneous performance including the initial efficiency and the initial resistance, rarely investigating the performance with the continuous mass loading. As the resistance gradually builds up with the mass loading of particles, the rate of increase with the continued loading may be more important in practical situations. In general, fluffy structures with large pores are preferred as they promote the depth filtration, leading to a lower resistance with the continued mass loading. Noticing that a nanofabric, commonly forming a compact structure, can be problematic to achieve a low resistance, Fan et al. [101] fabricated a fluffy, cotton candy-like structured web with fibers with ribbon morphology via electrospinning of the zein protein, using a mixture of different solvents that make the zein protein metastable. The ribbon-like fibers were curved to produce large pores within the web, giving a high air permeability and filtration capacity.

### 4.2. Hybrid Morphology and Layered Design of Fibrous Assembly

Hybrid fiber webs with multi-components and various structures have been extensively studied [84,85,93,94,103,106,107,108]. A hierarchical double layer structure with nanofibers and porous microfibers of poly(lactic acid) was fabricated by electrospinning; in this process, the pores of microfibers were designed by varying the relative environmental humidity, and the fiber size was controlled with the electrolytes such as LiBr in the pre-spinning solution [108]. The mass ratio of 1:5 of nanofiber:porous microfiber was reported to be optimum in giving a low resistance and high filtration efficiency in this hierarchical structure [108]. As a facile approach of making a binary structure with microfibers and nanofibers, a multi-spinning process was applied. Zhang et al. [93] made an intermingled binary structure with polysulfone (PSU) microfibers and polyacrylonitrile (PAN) nanofibers via multi-jet electrospinning, and the web had bimodal distribution of pore size with an excellent quality factor.

The main purpose of making a hybrid, multi-component structure is to improve the dust holding capacity and service life by reducing the resistance. The reduced resistance is also translated as breathing comfort for respirator users and energy saving for the HVAC system. Liu et al. [103] fabricated a bi-component spunbond web, employing polypropylene as a core and polyethylene as a sheath material. In this study, process parameters were controlled such as quenching temperature and drawing air pressure to produce a porous fluffy web [103]. The resulting spunbond web with through-air bonding showed an improved dust holding capacity compared to the conventional meltblown filter web or electrospun web. As another approach of making a hybrid structure, free surface electrospinning with a dual hopper was employed [106]. From one hopper, scaffold nanofibers of ~139 nm were produced, and from the other hopper, microspheres with nanofibers ~80 nm were produced. The fibers and spheres were intertwined and formed a 3D ternary structure, giving a low resistance filter media [106].

**Figure 6:**
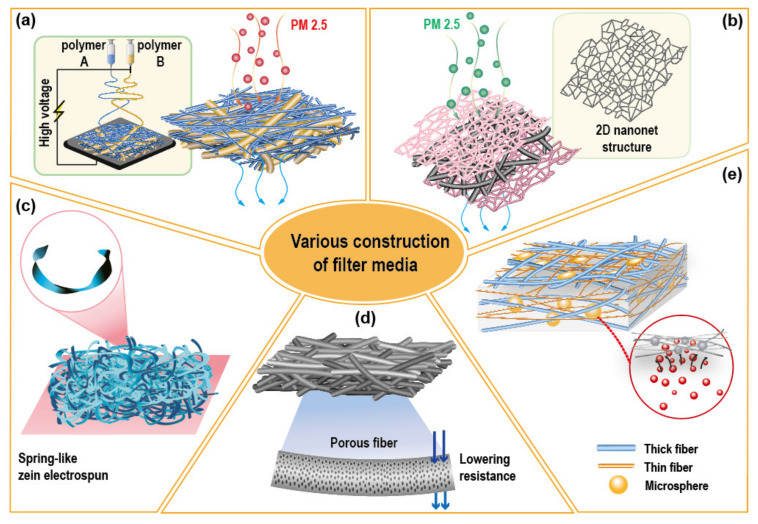
Illustration of various morphology and construction of fibrous assembly applied as filter media. (**a**) Mix of micro and nanofibers by dual electrospinning [63,85,93]; (**b**) Two-dimensional (2D) nanonets with three-dimensional (3D) fibers [84,85,86]; (**c**) Ribbon-structured fibers for constructing a fluffy web [65]; (**d**) Porous fibers [73]; (**e**) Hybrid structures with microfiber, nanofiber, and microspheres [84,93,106].

A sandwiched structure with dual morphology was fabricated via sequential electrospinning [84]; in this study, PAN bead-on-string fibers were sandwiched between polyamide (PA) nanonet layers. The produced structure had a stable cavity supported by the bead-on-string layer, and exhibited robust mechanical properties with a high quality factor. From the study by Zhang et al. [85], the dual layered structure was evolved to a triple layered structure for the multilevel physical capture of particles. The gradual variation of fiber diameter and pore size (~2.2, ~0.6, ~0.27 μm, respectively) was intended in this study, and the triple layers of microfiber, nanofiber, and nanonet morphologies were constructed by the sequential electrospinning. Notably, the electret charges of the produced web were eliminated by isopropanol to account for only the mechanical filtration efficiency of the web. The multi-layered hybrid construction has been suggested as a powerful approach for improving the quality factor and dust holding capacity, enabling the depth filtration with a multi-level capture of various sized particles [84,85,108,109]. Various morphological variations of filter media that have been investigated in recent studies are illustrated in Figure 6.

### 4.3. Modeling and Simulation for Particle Capture Behavior

Filtration modeling and simulation have been recently adopted to interpret the particle capture behavior of filter media [31,42,82,85,86,104,124,125,126]. The predictive capabilities of simulation depend on the accurate representation of filter construction. Micro computed tomography (μ-CT) can be employed to scan the 3D architecture of filter web, but the fiber size of several micrometers range is often too small to be precisely captured by this technique. Another approach was to use the scanning electron microscopy (SEM) image to capture the 2D image of surface fibers, then to construct the fibrous assembly in depth layers to extract the 3D web formation [127,128]. For the realistic 3D representation of the web, a 3D shape of each fiber section was created by rotating with a particular diameter circle along the centerline, and then by stacking the individual nanofiber layers on each other with a particular space between them [62].

More recently, modeling modules such as FiberGeo and FilterDict have been used to extract the representative domains for filter construction to simulate the filtration process [85,86,104]. The resistance of the modeled filter was calculated to investigate the pressure distribution throughout the bulk of the filter material [82,85]. Moreover, the particle capture was simulated by tracking the particles across the filter media under a specific fluid condition. By feeding the particles with various sizes into the filter layers, the effect of fiber size and layer construction on depth filtration was investigated [85]. Likewise, the modeling and simulation technique can be applied to design high-performing filters, enabling the predictive engineering for superior filtration and prolonged service life. In this way, the modeling technique would save the massive load of experiments at least partially.

## 5. Multi-Functional Filter Development

### 5.1. Antimicrobial Filters

While ambient microbial aerosol and droplets can be filtered by the particulate filtration mechanism [24,129,130,131,132], more research attention was drawn to develop antimicrobial filters as a facile protective strategy [133,134,135,136]. The antimicrobial fibers are commonly based on the bactericidal effect of incorporated agents that disrupt the cell membrane. As antimicrobial agents, metals such as Ag, Au, and Cu, and photocatalytic metal oxides such as TiO_2_, ZnO, and MgO are commonly employed [134,135,137,138,139,140,141]. When the metal type is used, the metal ions and the reactive oxygen species (ROS) are released from the metal oxidation, and they cause cell death by damaging the proteins of membranes [138,142,143]. Silver has been widely employed as an antimicrobial agent [134,135,137,138,142,143]; when the silver nanoparticle-decorated silica hybrid particles were incorporated in the air filter material [143], the produced web showed the prompt and synergistic antibacterial activity against both Gram-negative (*E. coli*) and Gram-positive (*S. epidermidis*) bacteria.

The metal oxide of TiO_2_ has also been widely applied as an antimicrobial agent due to low cost and low toxicity. When TiO_2_ absorbs the UV light, the electron on the valence band overcomes the band gap and moves to the conductive band, generating electron hole pairs [144]. During this process, negative electrons generate O^2−^, and the positive electron holes generate the hydroxyl radicals which attack and kill the bacteria and viruses [145]. Naruporn et al. [146] fabricated a honeycomb structure of the TiO_2_-decorated hydroxyapatite composite (HA/TiO_2_), and this membrane showed 90–95% antibacterial efficacy against *E. coli* and *S. aureus* when exposed to UV light for 6 h. Recently, metal organic frameworks (MOFs) have been applied as a bactericidal agent, where ROS is generated from metal ions to kill bacteria [147]. ZIF-8, MOF-199, and Ag-MOF were tested by incorporating in the cellulose filters, and the incorporated filter showed ~99.9% bactericidal efficacy against *E. coli* [148].

Other than the bactericidal effect, anti-adhesive surfaces have been studied as to circumvent the adhesion of bacteria. The adhesion of bacterial cells on the flat surface is known to be mainly governed by the van der Waals interaction between the surface and the bacterial membrane. For fibrous surfaces, the adhesion mechanism is very complex, as the multiple factors including material chemistry, material morphology, cell morphology and chemistry need to be considered [149]. The criteria of surface properties for prevention of bacterial adhesion is not conclusive, but it has been generally reported that the moderately hydrophobic or moderately hydrophilic surfaces with a water contact angle in the range of 54 ~ 130° were subject to greater bacterial adhesion [150,151]. From the study where the wettability was controlled by the surface energy and nanoscale roughness, the superhydrophobic surface with nanoscale roughness showed a very low bacterial adhesion because the attachment of bacteria cell on the surface was prevented by the presence of trapped air in the nanoscale protrusions [150,152]. Furthermore, a superhydrophilic surface also showed low bacterial adhesion, because the tightly bound water layer caused the repulsive interaction between the bacteria and the surface [150,152]. While rough surfaces would afford favorable sites for bacterial adhesion with higher specific surface area, the effect of surface topography on cell adhesion is not conclusive as its interaction with bacterial morphology is varied [153,154,155]. More research is necessary to find the explicit criteria of surface topography and chemistry influencing the bacterial adhesion.

### 5.2. Gas and Vapor Adsorption

Toxic gases such as carbon monoxide (CO), nitrogen oxides (NO_X_), sulfur oxides (SO_x_), hydrogen sulfide (H_2_S), and volatile organic compounds (VOCs) are common airborne threats for public health [156]. Filtration of gaseous matter is commonly performed by microporous sorbents and particles [156]. Activated carbons (ACs) have been exclusively used to adsorb gas and vapor [12,13], and depending on the type of gases, ACs can be modified with reactive compounds such as metal salts, acids, and amines to enhance the adsorption of different chemicals [11,157].

More recently, the metal organic framework (MOF) has drawn attention as a gas adsorbent, with its high porosity, large surface area, and thermal stability [79,80,148,158,159]. MOF is composed of a cluster of metal ions and organic linkers, where the linker length and the chemical functionality can be tailored [160]. Britt et al. [158] reported the possibility of selective adsorption of MOFs for different gases; in this study, MOF-5, IRMOFs-3, MOF-74, MOF-177, MOF-199, and IRMOF-62 were tested against sulfur dioxide, ammonia, chlorine, tetrahydrothiophene, benzene, dichloromethane, ethylene oxide, and carbon monoxide. When MOF was applied to the filter media, the chemical functionality of MOFs, open pore size, and the surface charge affected the capture of gas and particulate matters. For example, Bian et al. [79] used ZIF-67 on a PAN/cobalt (II) acetate electrospun filter and the produced web showed over 99% for PM_2.5_ filtration efficiency and 84% for formaldehyde removal efficiency. Zhang et al. [80] embedded ZIF-8, UiO-66-NH_2_, Mg-MOF-74, and MOF-199 within PAN, PS, and PVP fibers, and the resulting filter fibers showed high capturing efficiency against both particulate matters (PM_2.5_ and PM_10_) and SO_2_ from the SO_2_/N_2_ mixture. Other than the MOFs, metal oxide particles such as titanium dioxide (TiO_2_), zinc oxide (ZnO), and aluminum oxide (Al_2_O_3_) have also been used as detoxifying agents in the filters [72,137,161,162,163,164].

The addition of gas adsorbent particles to filter media may lead to an increase of resistance. Thus, for effective dual filtration for gas and particles, the filter needs to be properly designed so that the pores of the filter media are not clogged too early [27].

### 5.3. Reusable and Washable Filter

As the disposable filters may add an environmental burden [165], the washable filter has been developed to extend the service life of filter materials. As the washing procedure can lead to the charge decay of electrostatic filters, the washing is not generally recommended [166]. As a way to overcome the charge decay, application of tribocharged media has been investigated [166,167,168]. Tribocharging is generated when the materials with different triboelectricity contact each other. As the triboelectric generator has the ability to convert the mechanical energy into the electrical energy when two objects come into contact, the tribocharging can be regenerated when needed. Bai et al. [166] fabricated a washable multilayer triboelectric air filter with polytetrafluoroethylene (PTFE) and nylon fabrics. The triboelectricity of the developed material could be regenerated after washing, and the filter maintained ~96% filter efficiency against PM_2.5_ after five times of washing.

As another approach to designing a washable filter, Zhang et al. [169] used an ionic liquid polymer (ILP) on the melamine-formaldehyde (MF) sponge membrane. ILP had a high electrostatic potential, and with power supplied, the ILP-coated filter showed an effective filtration efficiency. In this invention, the filter itself acted as an electrostatic conductor and reservoir of dust. The filter can be cleaned and reused until 10 times of washing, as long as the coated material functions upon the power supply.

Several studies investigated the reusability of filter materials after cleaning [78,170]. However, most of the work examined the efficiency of individual filter materials they developed, and the results cannot be generalized. As the reusability would be dependent on the extent of charge dissipation and mechanical deformation of filters, the factors including charging process, polymer properties, and physical structure of filter material are all important. Further studies are needed on the reusability of filters with the varied material properties and maintenance methods.

### 5.4. Environmentally Sustainable Filters

Common filter materials are PP and polyethylene terephthalate (PET), and they are hardly biodegradable. With the growing concern on the increasing amount of filter waste, filter development using biodegradable materials is gaining more attention. Soy bean protein is one of the actively used biomaterials with its availability as low-cost and processability. In many studies, soy protein was electrospun to form nanofibers, and the produced nanofibers demonstrated good filtration performance against solid particles and smoke [101,171,172,173]. Souzandeh et al. [171] fabricated a nanoweb with soy protein isolate and poly(vinyl alcohol) (PVA), and reported that the functional groups of protein was beneficial in attracting airborne particles and smoke.

Other protein materials such as silk, gelatin, and chitosan have been investigated for their effectiveness as a filter material [95,174,175]. Silk fibroin nanofibers showed 98.8% filtration efficiency for PM_2.5_ with a relatively lower resistance [95]. The gelatin nanofibers were electrospun onto the cellulose paper, and their layers were used as a gas filter, which exhibited filtration performance >80% for various toxic chemicals including HCHO, CO, SO_2_, and VOC_S_ [175]. Chitosan nanocomposite blended polyvinyl alcohol (PVA) membrane showed >95% for PM removal and >91% for antibacterial efficiency against *E. coli* and *S. aureus* [174]. Polylactic acid (PLA) is a bio-derived and biodegradable polyester, and its applicability as a filter material has been studied [97,108,111,112]. Likewise, PVA was studied as a biodegradable filter material [176].

In addition to employing the bio-derived polymers, waste recycled filters have also been developed. Zulfi et al. [100] recycled the acrylonitrile butadiene styrene (ABS) waste into a nanofiber web via electrospinning, and applied it as a filter. The ABS membrane was processable with solvents of dimethylacetamide, dimethylformamide, and tetrahydrofuran, and the resulting webs exhibited PM_2.5_ filtration efficiency greater than 95%. Polystyrene filter webs were made from the recycled waste of high-impact polystyrene (HIPS) and expanded polystyrene (EPS), and the recycled webs showed the filtration efficiency greater than 99.99%, which was comparable to the commercial high-efficiency particulate air (HEPA) filter [98,99].

In addition to the efforts using biodegradable and recycled resources, the development of an environmentally responsible process is gaining great attention. While the electrospinning process is regarded as a promising method for manufacturing effective filter media, potential risks associated with harmful organic vapor generated from the process need to be carefully monitored [177]. As the ‘green electrospinning’ method, solvent-free electrospinning [178,179,180] and electrospinning with non-toxic solvents [180,181,182,183,184] are being studied. Electrospinning with an aqueous pre-spinning solution can easily achieve the non-toxic manufacturing, but its vulnerability to moisture remains as a drawback. With such a restraint, the thermal cross-linking method has been employed as a post-treatment of electrospinning [181,182,183]. Thermal cross-linking enhanced the thermal and chemical stabilities of polymeric membrane by forming a stable three-dimensional network structure [185], and produced an efficient filter media. In many studies [181,182,183], PVA was used as a water-soluble polymer in pre-spinning solution, and esterification of PVA was performed by the thermal cross-linking, producing a high efficiency filter material (>99%). Moreover, photo cross-linking via ultraviolet (UV) irradiation was conducted as another way of ‘green electrospinning’ [184], where the photo cross-linked PVA/chitosan nanofibers showed high filtration efficiency (>99%). Much endeavor is to be made to reduce the environmental impacts by the eco-friendly manufacturing process and by filter development with biodegradable, recyclable, or reusable resources.

## 6. Summary and Outlook

With increasing air pollution, there is ramping attention on the filtration devices. A number of studies have been conducted in the past ten years to explore the solutions for serious air pollution, especially in the Asia region. The main constituents of airborne pollutants include particulate matters of solid particles, liquid aerosol, microbial aerosol/droplets, gas, and vapor. In this paper, research efforts on the fiber-based particulate filters were reviewed with a focus on the following: (1) Understanding the filtration mechanism in relation to environmental factors such as face velocity, aerosol type, heat, humidity, and solvent exposure; (2) developing novel filter materials for enhanced filtration against various pollutants; (3) designing a breathable filter by lowering the pressure drop of filter media and filter assembly; (4) applying modeling and simulation techniques to understand the filtration process and to predict the performance; (5) exploring reusable and eco-friendly filter materials.

The overall mechanical filtration efficiency is the result of a combined effect of interception, inertial impaction, diffusion, and gravitational settling, and those mechanisms are affected by the particle size and the air velocity. There exists the most penetrating particle size (MPPS) range where the particle capture is the least effective, and the electrostatically charged filters significantly improves capturing particles especially in MPPS. An electret filter as opposed to a mechanical filter generally gives a higher quality factor, as the electrostatic attraction contributes as an additional capture mechanism. With such benefits of electret filters, long-lasting charges are the key to sustain the filtration efficiency.

Efforts have been made to increase the surface potential of electret filters and to enhance the long-term service performance, by exploring various material options and process techniques. The electrostatic charges of filter materials are influenced by environmental conditions such as temperature, humidity, and presence of organic vapors, and also by the polymer properties such as electric conductivity and moisture absorption capacity. In general, the polymers with low dielectric constants and low moisture absorption were advantageous for retaining charges longer. Filtration efficiency was more rapidly deteriorated by the oily aerosol than the solid aerosol, because the oily aerosol quickly spread on the charged fibers then masking the charged sites. As an easy solution to this, an anti-wetting treatment of fibrous media was conducted to delay the wetting of oily liquid.

As much efforts were made to lower the resistance of filter media by controlling the fiber size from nano to micrometer scale, and designing large pores and space between the fibers. The hierarchical layer design and multi-component web structure were rather effective in delaying the clogging of filters. Multifunctional filters that show antimicrobial effect and/or gas and vapor adsorption have been studied with great interest. Along with the emphasis on the environmental sustainability, reusability of filters and environmentally sustainable filters have been investigated. In addition to material development, modeling and simulation techniques have been adopted to interpret the particle capture behavior; this technique would save a massive load of experiments in the future.

With the sporadic occurrence of epidemic outbreaks such as COVID-10, SARS, H1N1 influenza, the role of air purifying respirators became an essential to the public. Much work has been done in maturing the research field of air filtration, but there is much to be done in the future. So far, most of the research has been focused on developing novel materials by the lab-scale processes, without much consideration of scalability. The scalable process needs to be further investigated to realize the exploratory concept materials. Moreover, in most of the research, the developed material was evaluated for the instantaneous performance in demonstrating the superior performance of the newly developed materials. As the filtration performance should sustain during the continuous loading of pollutants in real application, the performance evolvement with the continued exposure to pollutants needs to be further investigated.

While filtration performance against bacterial aerosol and droplets has been examined, rarely has the filter been evaluated for virus filtration, mainly due to the limited availability of test setup with the proper biosafety. Test methods that simulate the virus filtration need to be developed. Moreover, as novel materials are being developed, the toxicity of functional agents should be carefully analyzed. Together with the functional performance, comfort properties such as breathability and vapor transmission need to be prudently examined for assuring the protection with the minimal physiological burden.

## Figures and Tables

**Table 1:** Airborne pollutants [3,4,5,6,7].

Types of Pollutants		Size Range (μm)	Protective Tool
Solid/liquid particulates	Solid particle [3]	Varied	Particulate filter
	Liquid aerosol [4]	Generally ≤ 5	
	Liquid droplet [4]	Generally > 5	
Bioaerosol	Bacteria [5,6]	0.5–2	Particulate filter, antimicrobial
	Virus [5,6]	0.02–0.3	
Gas and vapor	Volatile organic compound (VOC) [7]	< 0.001	Gas adsorbent (activated carbon, etc.)
	Gaseous matter (SOx, NOx, etc.) [3,7]	< 0.01	

**Table 2:** Design strategies for advanced particulate filter materials.

Purpose	Fabrication Method	Ref.
Enhancing charge stability and electrostatic filtration	Using polymer with low dielectric constant Combining high electrical resistive webs with charge enhancing materials Incorporating magnetic particles to fibers Hybridizing the materials with different dielectric constants	[41,54,62,75] [41,45,55,64] [54,66,67,68,69,70,71,72,73,76] [33,34,41,55,77]
Enhancing mechanical filtration performance	Anchoring MOFs on nanofibers Combining nanonet and nanofiber	[78,79,80] [42,81,82,83,84,85,86,87]
Stabilizing filtration performance under humid or high temperature condition	Stabilizing surface potential of filter membrane Using materials with high thermal stability	[64,65,88] [89,90,91,92]
Enhancing filtration efficiency against oily aerosol	Lowering the surface energy via fluorination	[24,30,33,74]
Lowering resistance and facilitating depth filtration	Layering with large and small fibers Making hybrid structure with beads and fibers Making fluffy web Fabricating 2D nanonet structure with nanowires Making functional core shell structure fibers Designing 3D ternary structural membrane Constructing sandwich as a multi-layer filter	[63,93,94,95,96] [71,84,97,98,99,100] [87,101,102,103] [42,81,82,83,104] [88,103] [85,93,104,105,106] [31,63,84,85,107,108,109]
